# Sleep Quality and Its Contributing Factors Among Patients With Obesity: A Cross-Sectional Study

**DOI:** 10.7759/cureus.74038

**Published:** 2024-11-19

**Authors:** Hasan A Saeed, Ali H Mohsen, Ahmed T Alqayem, Sadiq H Hasan, Mohamed M Hasan, Husain A Alzeera

**Affiliations:** 1 College of Medicine, Wenzhou Medical University, Wenzhou, CHN; 2 College of Medicine, Zhejiang University, Hangzhou, CHN; 3 Department of Emergency Medicine, Salmaniya Medical Complex, Manama, BHR

**Keywords:** bmi, comorbid conditions, lifestyle factors, obesity, saudi arabia, sleep disturbances, sleep quality

## Abstract

Background: Obesity is a major public health issue associated with a range of comorbid conditions, including sleep disturbances. Poor sleep quality is common in individuals with obesity, yet the factors contributing to this relationship remain underexplored, especially in non-Western populations. This study aimed to investigate sleep quality and its contributing factors among patients with obesity in the eastern region of Saudi Arabia.

Methods: A cross-sectional study was conducted in multiple healthcare centers in the eastern region of Saudi Arabia. Two hundred adults (aged 18-65 years) with obesity (BMI ≥30 kg/m²) were recruited through convenience sampling. Data were collected using a structured questionnaire that assessed demographics, sleep patterns, lifestyle factors (e.g., physical activity, dietary habits, electronic device use), and comorbid health conditions. Sleep quality was self-reported using a four-point scale. Statistical analyses, including descriptive statistics and chi-square tests, were used to identify relationships between BMI and sleep quality.

Results: The mean age of participants was 42.5 years (SD = 12.3), with 56% female. Participants reported an average sleep duration of 5.8 hours per night (SD = 1.3). Over 50% of participants experienced poor sleep quality, and 64% reported symptoms of sleep apnea. Increasing BMI was associated with poorer sleep quality, with those in the highest BMI categories (BMI >42 kg/m²) reporting the worst sleep outcomes. Lifestyle factors such as physical inactivity (75%) and caffeine consumption (60.5% within six hours of bedtime) were also significantly associated with poor sleep quality.

Conclusions: Obesity is strongly associated with poor sleep quality in this cohort, with higher BMI and unhealthy lifestyle factors contributing to sleep disturbances. Interventions targeting weight management, physical activity, dietary habits, and sleep hygiene are essential for improving sleep quality and overall health in obese patients. Future research should explore the causal mechanisms between obesity and sleep disturbances and evaluate the effectiveness of integrated obesity and sleep interventions.

## Introduction

Obesity has reached epidemic proportions worldwide and is recognized as one of the most significant public health challenges of the 21st century. According to the World Health Organization, more than 1.9 billion adults were overweight in 2016, with over 650 million of them classified as obese. In Saudi Arabia, the prevalence of obesity has been rising steadily over the past decades, with recent estimates indicating that approximately 35% of the adult population is obese [1-3]. Obesity is a multifactorial condition that results from a combination of genetic, environmental, and behavioral factors, and it is associated with a wide range of comorbidities, including type 2 diabetes, cardiovascular diseases, hypertension, and certain cancers [2,4].

Beyond its well-known physical health consequences, obesity has also been linked to various sleep disturbances, which can exacerbate the burden of obesity-related health problems. Despite the growing recognition of the impact of obesity on sleep, there remains a lack of comprehensive research exploring the specific relationship between obesity and sleep quality, especially in non-Western populations [2-5].

Sleep is a critical physiological process essential for overall health, affecting cognitive function, emotional well-being, and physical health. Adequate sleep, typically defined as seven to nine hours per night for adults, is necessary for optimal brain function, metabolic regulation, immune function, and cardiovascular health. Sleep disturbances, including difficulty falling asleep, frequent awakenings, and feelings of non-restorative sleep, are common in modern societies and are associated with a variety of health problems [1-5].

Sleep quality is increasingly being recognized as an important indicator of health. Chronic sleep deprivation and poor sleep quality have been linked to a wide range of negative outcomes, including impaired cognitive performance, increased risk of cardiovascular disease, metabolic dysregulation, and mental health disorders such as depression and anxiety [5-8]. In individuals with obesity, poor sleep quality may contribute to a cycle of weight gain, as sleep deprivation is associated with increased hunger and changes in food preferences. Additionally, sleep disturbances in obese individuals can be exacerbated by conditions such as obstructive sleep apnea, which is highly prevalent among individuals with high BMI [5,7].

Obesity has been shown to have a significant impact on sleep quality. The mechanisms underlying this relationship are complex and multifactorial. One of the most well-documented sleep disorders associated with obesity is obstructive sleep apnea (OSA), a condition in which the airway becomes partially or completely obstructed during sleep, leading to frequent awakenings and reduced oxygen saturation levels. OSA is particularly common in individuals with a BMI greater than 30 kg/m², and studies have shown that the prevalence of OSA increases with higher BMI [7-10].

In addition to OSA, obesity has been associated with other sleep disturbances such as insomnia, restless leg syndrome, and poor sleep quality. Several potential mechanisms may explain these associations. Excess adipose tissue, particularly abdominal fat, can place pressure on the diaphragm and impair respiratory function during sleep, leading to increased arousal and non-restorative sleep. Furthermore, adiposity-related metabolic changes, including insulin resistance and inflammation, may interfere with the body’s circadian rhythm and affect sleep regulation [10-13].

Lifestyle factors, such as physical inactivity, dietary habits, and electronic device usage, have also been implicated in the relationship between obesity and sleep disturbances. Sedentary behavior and lack of physical activity are well-known contributors to poor sleep quality, and the use of electronic devices, particularly in the evening, can disrupt circadian rhythms and negatively impact sleep onset. Moreover, dietary patterns high in processed foods, sugar, and caffeine are associated with poorer sleep outcomes, and these habits are more prevalent in individuals with obesity [5-9].

While the relationship between obesity and sleep quality is well-documented in Western populations, there is a paucity of research examining this association in the Middle Eastern and Saudi populations. Understanding how obesity and sleep disturbances interact in this specific cultural context is crucial for developing region-specific interventions to improve sleep and overall health. Furthermore, much of the existing literature focuses on clinical populations, such as those diagnosed with sleep apnea, and less attention has been paid to the broader population of obese individuals who may not have been formally diagnosed with sleep disorders but still experience significant sleep disturbances.

## Materials and methods

Study design and participants

This cross-sectional study was conducted in multiple healthcare centers in the eastern region of Saudi Arabia. A total of 200 adult participants, aged 18-65 years, with obesity (defined as a BMI ≥ 30 kg/m²) were recruited through convenience sampling. Participants were selected from both urban and suburban clinics, ensuring a diverse representation of gender, age, and socio-economic background. Exclusion criteria included individuals with severe psychiatric disorders, acute medical conditions, or those who had undergone bariatric surgery.

Data collection

Data were collected through a structured questionnaire administered during face-to-face interviews by trained research assistants. The questionnaire was designed to capture information on participant demographics, sleep patterns, lifestyle factors, and comorbid health conditions. Demographic data included age, gender, marital status, employment status, and BMI. Participants were asked about their average sleep duration, difficulty falling asleep, frequency of night awakenings, and overall sleep quality, which was rated as excellent, good, fair, or poor. Lifestyle factors, such as physical activity frequency, dietary habits (including caffeine consumption), and electronic device usage before sleep, were also assessed. Finally, participants were questioned about the presence of comorbid conditions such as hypertension, diabetes, depression, and sleep apnea.

Measurement of variables

BMI was calculated based on self-reported height and weight. Participants were categorized into four BMI groups: 28-32 kg/m², 33-37 kg/m², 38-42 kg/m², and >42 kg/m². Sleep quality was assessed through self-reported ratings, which classified participants' sleep as excellent, good, fair, or poor. Physical activity frequency was categorized as daily, three to five times per week, one to two times per week, or rarely/never. Dietary habits were assessed based on the frequency of caffeine consumption and the intake of high-fat or high-carbohydrate foods. The presence of comorbid conditions was assessed through self-reporting of prior diagnoses by healthcare providers.

Statistical analysis

Data were analyzed using Statistical Product and Service Solutions (SPSS, version 26.0; IBM SPSS Statistics for Windows, Armonk, NY). Descriptive statistics were used to summarize demographic characteristics, sleep quality, and lifestyle factors. Continuous variables were presented as means with standard deviations (SD), while categorical variables were expressed as frequencies and percentages. Statistical significance was set at p < 0.05.

## Results

Participant demographics

The study included 200 participants, with a mean age of 42.5 years (SD = 12.3), ranging from 18 to 65 years. The majority of participants were female (56%), followed by males (42.5%), and a small percentage identified as other genders (1.5%). The average BMI of participants was 34.8 kg/m² (SD = 6.7), with a range from 28 to 51 kg/m². Most participants were married (59%), 29% were single, and 12% were divorced or widowed. Regarding employment status, 72% were employed, 18% were unemployed, and 10% were retired (Table 1).

**Table 1:** Participant demographics (N = 200) This table presents the demographic characteristics of the study participants, including age, gender, BMI, marital status, and employment status.

Variable		Mean (SD)/n (%)
Age (years)		42.5 (12.3)
Gender	Male	85 (42.5%)
	Female	112 (56%)
	Other	3 (1.5%)
BMI (kg/m²)		34.8 (6.7)
Marital Status	Single	58 (29%)
	Married	118 (59%)
	Divorced/Widowed	24 (12%)
Employment Status	Employed	144 (72%)
	Unemployed	36 (18%)
	Retired	20 (10%)

Sleep patterns among participants

The average reported sleep duration was 5.8 hours per night (SD = 1.3), with a range of three to eight hours. In terms of difficulty falling asleep, 17.5% of participants reported never having trouble, 43.5% occasionally experienced difficulty, 29% often had trouble, and 10% always struggled to fall asleep. Regarding night awakenings, 48.5% of participants woke up one to two times during the night, 32.5% woke up three to four times, and 14% reported more than four awakenings. Sleep quality was generally fair to poor: 11% of participants rated their sleep as excellent, 39.5% as good, 34% as fair, and 15.5% as poor (Table 2).

**Table 2:** Sleep patterns among participants (N = 200) This table summarizes participants' sleep patterns, including average sleep duration, difficulty falling asleep, frequency of night awakenings, and self-reported sleep quality.

Sleep Variable		Mean (SD)/n (%)
Sleep Duration (hours/night)		5.8 (1.3)
Difficulty Falling Asleep	Never	35 (17.5%)
	Sometimes	87 (43.5%)
	Often	58 (29%)
	Always	20 (10%)
Night Awakenings	1-2 times	97 (48.5%)
	3-4 times	65 (32.5%)
	More than 4 times	28 (14%)
Sleep Quality	Excellent	22 (11%)
	Good	79 (39.5%)
	Fair	68 (34%)
	Poor	31 (15.5%)

Lifestyle factors and behaviors influencing sleep

Lifestyle factors were found to influence sleep patterns. Only 24.5% of participants engaged in physical activity at least three times a week, while 79% used electronic devices (e.g., phones, TVs) within two hours of bedtime. Furthermore, 60.5% of participants consumed caffeine within six hours before sleep. Diets varied: 39% reported following a balanced diet, 46.5% followed a high-fat or high-carbohydrate diet, and 14.5% had an irregular or inconsistent diet (Table 3).

**Table 3:** Lifestyle factors and behaviors influencing sleep (N = 200) This table describes key lifestyle factors, including physical activity, caffeine consumption, use of electronic devices, and dietary habits that may influence sleep quality.

Variable	n (%)
Physical Activity (≥3 times/week)	49 (24.5%)
Caffeine Consumption (6h before sleep)	121 (60.5%)
Electronic Device Use (2h before sleep)	158 (79%)
Balanced Diet	78 (39%)
High Fat/Carb Diet	93 (46.5%)
Irregular Diet	29 (14.5%)

Prevalence of health conditions and sleep-related issues

Several health conditions were prevalent among participants. Nearly half (48.5%) were diagnosed with hypertension, 36% with diabetes, and 25.5% with sleep apnea. Additionally, 43% of participants reported symptoms of depression or anxiety, and 64% experienced snoring. When asked whether they felt rested upon waking, 15.5% reported always feeling rested, 48.5% sometimes, 24.5% rarely, and 11.5% never felt rested (Table 4).

**Table 4:** Prevalence of health conditions and sleep-related issues (N = 200) This table summarizes the prevalence of comorbid conditions such as hypertension, diabetes, sleep apnea, depression, and snoring, as well as participants' reports of feeling rested upon waking.

Variable		n (%)
Hypertension		97 (48.5%)
Diabetes		72 (36%)
Sleep Apnea		51 (25.5%)
Depression/Anxiety		86 (43%)
Snoring		128 (64%)
Feels Rested Upon Waking	Always	31 (15.5%)
	Sometimes	97 (48.5%)
	Rarely	49 (24.5%)
	Never	23 (11.5%)

Association between BMI and sleep quality

An association between BMI and sleep quality was observed (p = 0.02). Participants with a BMI between 28 and 32 kg/m² had the highest percentage of good or excellent sleep quality (58%) and a relatively lower percentage of poor sleep quality (18%). As BMI increased, poor sleep quality became more prevalent. In the 33-37 kg/m² group, 25% had poor sleep quality, while 51% had fair sleep quality. For participants with a BMI of 38-42 kg/m², 37% reported poor sleep quality, and only 3% reported good/excellent sleep. Among those with a BMI >42 kg/m², 50% experienced poor sleep quality, with no participants reporting good or excellent sleep (Table 5).

**Table 5:** Association between BMI and sleep quality (N = 200) This table shows the relationship between BMI categories and self-reported sleep quality, with participants categorized into poor, fair, and good/excellent sleep quality.

BMI Category (kg/m²)	Poor Sleep Quality (%)	Fair Sleep Quality (%)	Good/Excellent Sleep Quality (%)
28-32	12 (18%)	16 (24%)	39 (58%)
33-37	19 (25%)	39 (51%)	19 (25%)
38-42	26 (37%)	10 (14%)	2 (3%)
>42	17 (50%)	3 (9%)	0 (0%)

## Discussion

This cross-sectional study aimed to explore the sleep quality and contributing factors among patients with obesity in the eastern region of Saudi Arabia. Our findings reveal that poor sleep quality is prevalent among individuals with obesity, with nearly half of the participants reporting insufficient sleep duration and frequent disturbances during the night. The study also identified several factors, including obesity severity, physical activity levels, dietary habits, and comorbid health conditions, that significantly influence sleep quality. Furthermore, a clear association was observed between increasing BMI and poorer sleep quality, with individuals in the highest BMI categories (≥38 kg/m²) reporting the worst sleep outcomes.

Our results are consistent with a growing body of evidence linking obesity with poor sleep quality. Previous studies have shown that individuals with obesity often experience disrupted sleep, including difficulty falling asleep, frequent awakenings, and reduced restorative sleep [6-13]. In our study, participants with higher BMI levels (≥ 38 kg/m²) had the lowest self-reported sleep quality, which supports the notion that obesity may exacerbate sleep disturbances. This relationship could be attributed to factors such as obstructive sleep apnea, which is more prevalent in individuals with higher BMI, or the physiological impacts of excess weight on respiratory function and sleep architecture.

The association between increased BMI and poor sleep quality observed in our study is similar to findings from other populations, indicating that obesity is a significant risk factor for sleep problems across different regions and healthcare systems. This suggests that interventions targeting obesity management may also lead to improvements in sleep quality, further emphasizing the importance of integrated care approaches [7-10].

Our study found that lifestyle factors such as physical inactivity and the use of electronic devices before sleep were common among participants and were associated with poorer sleep quality. More than 75% of participants reported inadequate physical activity, and nearly 80% used electronic devices within two hours of bedtime. Both of these factors have been shown to disrupt sleep, with physical inactivity linked to reduced sleep quality and the blue light emitted from electronic devices known to interfere with circadian rhythms [10-13].

In addition, the presence of comorbid health conditions, particularly hypertension, diabetes, and depression, was prevalent among our participants. More than 40% of participants reported symptoms of depression or anxiety, and nearly 50% had hypertension. These conditions are well-known to exacerbate sleep disturbances and may be contributing to the observed sleep quality deficits in our cohort. Depression, in particular, is strongly linked to sleep disorders, with patients often reporting both difficulty falling asleep and frequent night awakenings [4-9]. The bidirectional relationship between sleep and mental health suggests that addressing psychological health may also improve sleep outcomes.

Dietary habits were another key factor influencing sleep quality in our study. Over 60% of participants reported consuming caffeine within six hours of bedtime, a habit known to negatively impact sleep by delaying sleep onset and reducing sleep depth. Additionally, nearly half of the participants followed a high-fat or high-carbohydrate diet, which has been linked to poorer sleep quality in multiple studies. Diets high in processed foods and low in micronutrients may interfere with the body’s ability to maintain restful sleep, possibly through mechanisms involving metabolic dysregulation or inflammation. These findings highlight the potential benefit of dietary interventions in improving sleep quality among patients with obesity. Incorporating nutritional counseling and promoting healthier eating habits could be valuable components of obesity management programs that also aim to improve sleep outcomes [5-11].

This study has several limitations that should be considered when interpreting the results. Firstly, the cross-sectional design limits the ability to establish causal relationships between obesity and sleep quality. Longitudinal studies are needed to determine whether improving sleep quality through weight management interventions leads to better long-term health outcomes. Secondly, the use of self-reported questionnaires to assess sleep quality and lifestyle factors may introduce recall bias, as participants might not accurately report their sleep habits or physical activity levels. Future studies could benefit from objective measures of sleep, such as polysomnography or actigraphy, to provide a more accurate assessment of sleep quality.

## Conclusions

In conclusion, this study highlights the significant association between obesity and poor sleep quality among patients in the eastern region of Saudi Arabia. Our findings demonstrate that obesity, particularly at higher BMI levels, is linked to a higher prevalence of sleep disturbances, including insomnia, frequent awakenings, and non-restorative sleep. Furthermore, lifestyle factors, such as physical inactivity, poor dietary habits, and comorbid conditions including hypertension and depression, contribute to the observed sleep quality deficits. Given the potential bidirectional relationship between obesity and sleep disturbances, addressing obesity through integrated interventions that target both weight management and sleep hygiene may improve health outcomes for affected individuals. This research underscores the importance of a comprehensive approach to managing obesity, which includes not only weight reduction but also the improvement of sleep quality, thereby enhancing the overall well-being of patients.
